# A Featureless Approach to 3D Polyhedral Building Modeling from Aerial Images

**DOI:** 10.3390/s110100228

**Published:** 2010-12-28

**Authors:** Karim Hammoudi, Fadi Dornaika

**Affiliations:** 1 Université Paris-Est, Institut Géographique National, 73 avenue de Paris, 94160 Saint-Mandé cedex, France; 2 Department of Computer Science and Artificial Intelligence, University of the Basque Country, Paseo de Manuel Lardizábal, 1, 20018 Donostia-San Sebastián, Spain; 3 IKERBASQUE, Basque Foundation for Science, 48011 Bilbao, Spain; E-Mail: fadi dornaika@ehu.es

**Keywords:** 3D building reconstruction, 3D city modeling, 3D polyhedral building model, aerial images, featureless image registration, Differential Evolution algorithm

## Abstract

This paper presents a model-based approach for reconstructing 3D polyhedral building models from aerial images. The proposed approach exploits some geometric and photometric properties resulting from the perspective projection of planar structures. Data are provided by calibrated aerial images. The novelty of the approach lies in its featurelessness and in its use of direct optimization based on image rawbrightness. The proposed framework avoids feature extraction and matching. The 3D polyhedral model is directly estimated by optimizing an objective function that combines an image-based dissimilarity measure and a gradient score over several aerial images. The optimization process is carried out by the Differential Evolution algorithm. The proposed approach is intended to provide more accurate 3D reconstruction than feature-based approaches. Fast 3D model rectification and updating can take advantage of the proposed method. Several results and evaluations of performance from real and synthetic images show the feasibility and robustness of the proposed approach.

## 1. Introduction and Motivation

**Figure 1.**
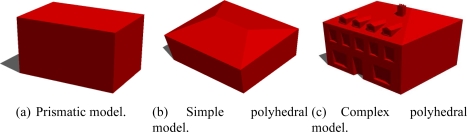
Examples of generic model representations. Three illustrations of the same building with different level of details (from low to high).

In the two past decades, the cartographic field has evolved significantly; mainly in order to provide a digital and 3D geometric description of urban environments in addition to 2D conventional paper urban maps. More precisely, some active work in the photogrammetric, remote sensing and computer vision communities is focused on the 3D building modeling approaches since the buildings constitute urban objects of great interest for the 3D city modeling. The 3D building modeling approaches are more and more developed due to the increasing needs of institutional and industrial applications in the civil and military contexts. The visualization of urban environments (e.g., virtual tourism), the urban planning, the site recognition (military applications) or the conservation of architectural work (cultural heritage) are some of the many applications requiring 3D building modeling approaches. For these reasons, several approaches are proposed across the literature and provide more or less accurate, detailed and adapted 3D building models according to the targeted applications. Globally, the proposed approaches tend to produce 3D building models with a quality closer to the physical reality. The prior knowledge of the urban areas under study (e.g., cities topology, environment densities, shape complexity, existing surveys, urban GIS databases) and the remotely sensed rawdata collected are very rich sources of information that can be used to develop sophisticated building modeling approaches. The 3D building reconstruction is a complex task due to the diversity of building shapes (e.g., architectural and contemporary buildings). The building facades usually have some microstructures (e.g., windows, doors) and the building roofs present some superstructures (e.g., chimneys, attic windows). The representations of 3D building models can thus be divided into three main categories (see Figure 1).

**Figure 2.**
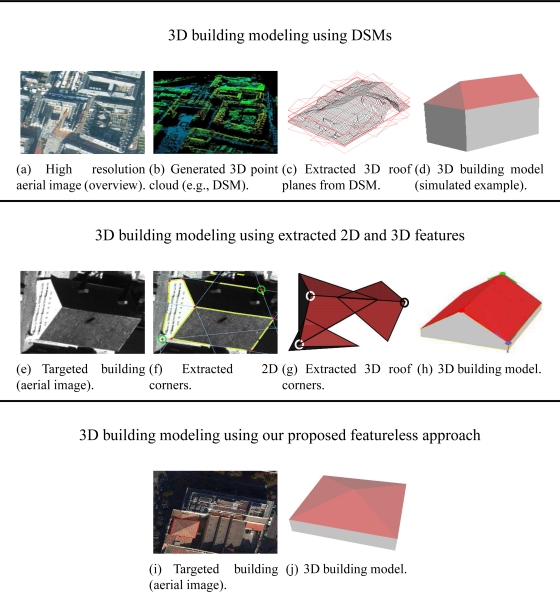
The upper part of this figure illustrates an example of 3D building modeling process using a DSM. The middle part of this figure shows image-based feature extraction and assembly. The lower part shows our proposed direct and featureless image-based approach. Figure 2(c) is retrieved from [1]. Figures 2(e), 2(f), 2(g) and 2(h) are retrieved from [3].

The complexity of 3D building models can be planimetric (complex polygonal ground footprint) as well as altimetric (e.g., heights variation). Aerial data are very useful for the coverage of large areas such as cities. In the literature, several aerial or satellite data-based approaches are proposed to extract 3D prismatic and polyhedral building models. The data usually employed as input to these approaches are either optical aerial or satellite images, aerial or satellite Digital Surface Model (DSM) or aerial 3D point clouds such as aerial LIDAR data (Light Detection And Ranging data). Some data samples usually employed are shown in Figure 2.

Figure 2 (Top) illustrates the building modeling using Digital Surface Models. Figure 2 (Middle) illustrates the building modeling using reconstructed geometrical features (e.g., 2D vertices and lines). Figure 2 (Bottom) illustrates our proposed featureless approach.

**Figure 3.**
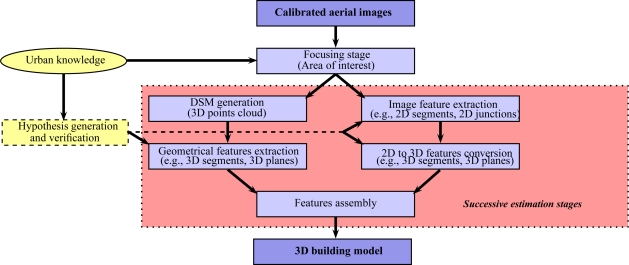
Flowchart diagram currently adopted by some image-based building modeling approaches. The diagram presents two paths conducting to 3D polyhedral building models. These two paths are illustrated by the first two rows of Figure 2.

**Figure 4.**
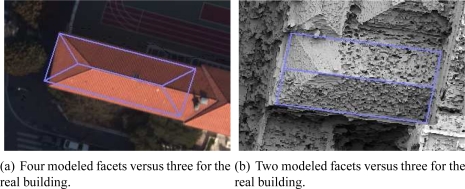
Some erroneous reconstructed buildings resulting from a known feature-based framework for massive building reconstruction (BATI-3D^®^ prototype software—a large scale building modeling pipeline developed at the French National Geographical Agency). The estimated 3D models are projected onto the image or DSM.

The flowchart of the two first strategies (image-based building modeling) is illustrated in Figure 3. In the first strategy, a DSM is generated or directly employed as input (e.g., [8]). An example of a very dense aerial DSM is shown in Figure 2(b). The succeeding stages consist of the use of the DSM as reference for the extraction of high level geometric features (e.g., 3D segments or 3D planes). The extracted features are finally assembled into a polyhedral building model using various optimization methods. However, these successive estimation stages inevitably introduce some inaccuracies that propagate from one stage to the next, which can affect the final 3D model. If these inaccuracies are large enough, then, one can note, that the obtained shape can be erroneous (e.g., see Figures 4(a) and 4(b)). In the second strategy, geometrical features are extracted from aerial images (e.g., 2D segments, junctions, corners, lines) and then converted into 3D features. The final polyhedral model is then estimated using these 3D features (e.g., Figure 2(g)). As in the first strategy, the extraction and matching stages inevitably affect the accuracy of the final 3D model. [2] and [3] are well-know references in the literature which respectively illustrate the two strategies described above.

The 3D building reconstruction of a full urban environment requires automatic or semi-automatic methods. The massive reconstruction approaches usually employ a feature extraction stage. However, this stage is very sensitive since it can induce some missed-detections, false alarms, under-detections or over-detections. To control these effects, the 3D building modeling approaches employ computer vision strategies. These strategies are regrouped into two paradigms. More precisely, the first paradigm is a bottoms-up scheme and consists in the assembly of geometric features without pre-existing knowledge of the sought model. The second paradigm, called top-down, exploits a library of models and searches the model that best fits with the input data (images, DSMs).

As previously mentioned, several approaches for 3D reconstruction of polyhedral building models currently employ as input Digital Surface Models (see Figure 2(b)). The classical DSMs are usually generated from calibrated aerial images by a multi-correlation based optimization process such as the graph cut optimization. The DSMs (derived data) are generally maps comprising only one value of altitude *z* for each ground location (*x, y*). These 2.5D maps can be considered as special 3D point clouds. However, the obtained 3D surface does not accurately model the physical surface especially at height discontinuities such as at roof and superstructure boundaries due to the correlation criterion used. Hence, the DSMs provide an approximated geometrical description of building surfaces and can be noisy. Other modeling approaches employ multi-source data, for example optical images combined with LIDAR data (e.g., [16]). Although less dense, LIDAR data can be employed in place of DSMs since they are both accurate (e.g., [9–13]).

### Paper Contribution

In this paper, we propose a direct and featureless approach for the extraction of 3D simple polyhedral building models from aerial images (Figure 2(a)). The novelty of our approach consists in the usage of a genetic optimizer which bypasses all the intermediary estimation/extraction stages previously mentioned. First results of our approach were presented at the MVA and ACIVS conferences, respectively in [17] and [18]. This paper presents a substantially extended version which describes in more detail the models as well as the core of the proposed methodology and processes.

We are interested in modeling residential buildings having simple polyhedral shapes and whose ground footprints are represented by quadrilaterals. We note that in most cases, these quadrilaterals are rectangles. However, this requirement is not a limitation to our approach. Indeed, any complex shape can be considered as a union of simple models with rectangular footprints.

The input data are calibrated aerial images. Hence, our research deals with the intermediary degree of generic modeling such as described in Figure 1(b). In our case, the proposed approach can be considered as a top-down scheme (model driven) in the sense that a library of parametric building models is employed. However, our top-down approach is not conventional in the sense that the 3D model estimation is direct and only uses image rawbrightness. Moreover, the exhaustive search for the best model is avoided. The proposed approach employs aerial images as illustrated in Figure 2(a). The building footprint (focus area) is selected by an operator in one aerial image. The building footprint could also be retrieved from a cadastral map (existing 2D map of building footprints) [1,4–7]. In this case, the 2D footprint is expressed in a georeferenced world coordinate system.

In this study, we are essentially focusing on the approaches producing polyhedral building models (as shown in Figure 1(b)) from a single source of data, namely the high resolution aerial images. These images represent the data type that is widely utilized. Consequently, several image-based modeling approaches are detailed in the following Section 2 (related work).

The rest of the paper is organized as follows. Section 2 describes various existing image-based approaches for 3D polyhedral building modeling. Section 3 presents the global strategy of the proposed approach. Section 4 describes the optimization process of the approach. Section 5 gives several intermediary results and evaluations of major steps.

## 2. Related Work

Many interesting building modeling approaches have been addressed in the literature for the reconstruction of 3D polyhedral building models (e.g., [23–34]). The intention here is to briefly describe some aerial-based approaches that are discerned by their optimization process, their global methodology or their efficiency.

In [1], Jibrini *et al.* propose a 3D polyhedral building modeling approach from a very high resolution aerial stereo-pair using a cadastral map. A cadastral map is a 2D ground map (detailed register) showing the parcel delimitations of each building. This standard 2D map is often used by governments for the annual taxation of their residents according to the size of their homes. Their proposed method is generic in the sense that it can be used to estimate the polyhedral shape of buildings without pre-existing knowledge about the real shape. Firstly, the corresponding volume of interest is set as an extrusion of the 2D footprint into 3D. This volume is then discretized and a correlation score is calculated for each voxel using a stereoscopic principle and a block matching method. The hypothesis of 3D planes are then detected using the Hough Transform (HT) weighted by the correlation score of each voxel. Several arrangements associated with these 3D planes inside the delimited volume are calculated. The research of admissible shapes will be equivalent to the research of maximal clicks in a compatibility graph. The last step selects the best admissible model by optimizing a term related to the data (compatibility between the model and the images) and a term of regularization related to the model complexity.

Taillandier *et al.* [2] present another generic approach that can be considered as an extension of the approach described in [1]. The reconstruction is directly achieved using a Digital Elevation Map generated from multi-view stereo images. A building is modeled by a polyhedral shape, without overhangs. The building boundaries are modeled by vertical walls. This proposed method is generic and allows the modeling of almost all building categories. For each building, an operator manually selects a focus area as well as a ground altitude. 3D planar features (horizontal, vertical and oriented planes) and 3D segments are then automatically extracted in this area. A 3D graph of arrangements is generated by the intersection of all the planes. After a graph simplification step, the search of admissible 3D models is proved to be similar to the search of maximal clicks. The model is finally selected using a Bayesian modeling method. In another work [6], Durupt and Taillandier have proposed operational approaches useful to adapt the generic algorithm to more realistic data. These approaches are mainly focused on the calculation of the 3D planes hypothesis.

In [8], Lafarge *et al.* propose an approach for the 3D building reconstruction in dense urban environments using high resolution satellite images. The approach employs a DSM and a set of parametric models. A marked point process is employed to automatically extract rectangular building footprints from the DSM. The best model parameters with a rectangular footprint are searched using pre-existing knowledge of classical models and their interactions. The data term minimizes the error between the models and the DSM. The model parameters associated with a block of buildings are obtained by searching the maximum a posteriori. This maximum is obtained using a RJMCMC method (Recursive Jump Monte Carlo Markov Chain) and a SA method (Simulated Annealing).

Fisher *et al.* present in [3] a model-based approach to the 3D building model extraction from aerial images. This proposed approach allows the reconstruction of various types of polyhedral buildings. The building parts are classified according to their roof types. The approach employs the extraction of low-level image features, the matching of these features according to building part models and the aggregation of the model into complete building models.

In [14], Jaynes *et al.* present a model-based approach to the automatic detection and reconstruction of buildings using aerial imagery. Optical aerial images are first segmented in order to detect the buildings. The corresponding DEM is employed to reconstruct the buildings. Each segmented DEM region is associated with a class of building roof shape either peaked, flat or curved. The segmented regions are extruded and fitted to the DEM by an optimization process. The segmented DEM region allows the decomposition of the building area into sub-area according to its roof shape. The final building model is obtained by the union of roof part models independently estimated. This strategy allows the reconstruction of a wide variety of polyhedral building models.

Zebedin *et al.* propose in [15] an approach for the automatic building reconstruction from aerial images. An approach is proposed to meet the need for realistic and accurate building models for virtual applications. Line features that characterize the height discontinuities are detected and combined with dense depth data providing the roof surface by using a global optimization process based on Graph Cuts technique. The proposed algorithm generates elegant building models. The approach has been analyzed and evaluated using ground truth data.

In [21], Tseng *et al.* propose a promising 3D building reconstruction approach that uses Genetic Algorithms (GA) for model-image fitting. The buildings are reconstructed piece by piece and each CSG feature (Constructive Solid Geometry) is fitted according to the edge pixels of aerial images. CSG boolean set operators are employed in order to combine building parts into a single building. The theory of the GA method for model image fitting has been analyzed and demonstrated in several examples.

Table 1 briefly presents some feature-based building modeling approaches available in the literature. The regrouped approaches demonstrate the high diversity of employed techniques in 3D building modeling from aerial images. The presented approaches propose building modeling advances at various levels of generalization, geometry, accuracy, and realism. The priority characteristic is guided by the targeted application. In our case, the main goal is (i) to improve the accuracy of 3D polyhedral building models using images, and (ii) to rectify the erroneous estimated shape of building model issues from certain feature-based approaches (as shown in Figure 4).

## 3. Problem Statement and Model Parametrization

In this section, we present our formulation of the problem and the adopted parametrization. In the previous section, we described several approaches that have been addressed in the literature. Here we state the characteristics of our approach.

**Figure 5.**
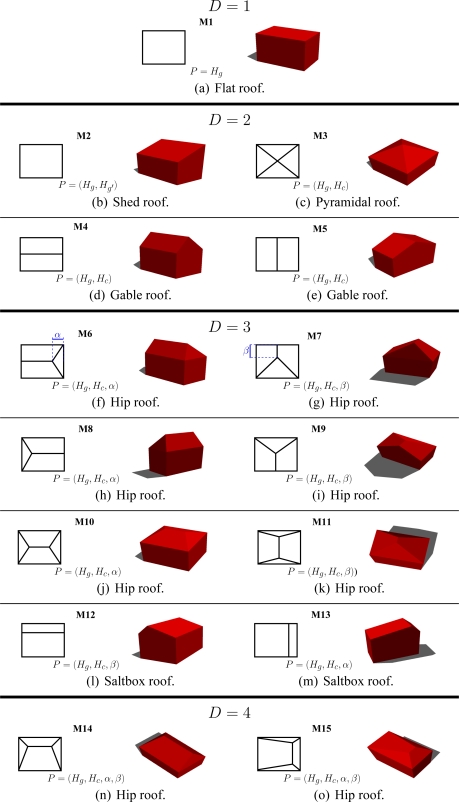
Samples of parametric building models *M* that can be reconstructed by our proposed featureless approach. Standard polyhedral shapes and their corresponding ground footprints are shown. *D* corresponds to the number of parameters. *P* denotes the model parameters. *H_g_* and *H_c_* correspond to the gutter height and the central line height. *α* and *β* respectively represent the horizontal and vertical recess as illustrated in blue.

**Figure 6.**
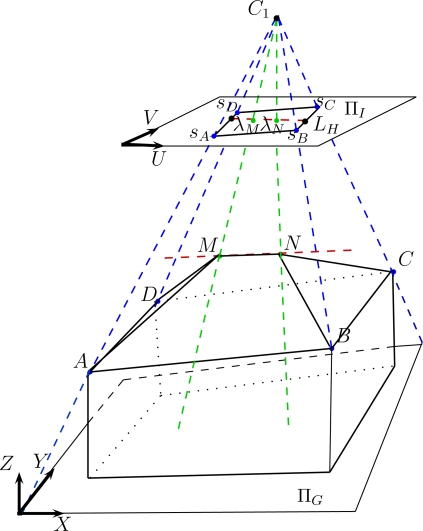
The adopted generic 3D polyhedral model. The multi-facet model (*i.e.*, deformable model) is parameterized by Equation (3). *s_A_*, *s_B_*, *s_C_*, *s_D_* correspond to the footprint vertices selected in the image plane Π*_I_* (master image). λ*_M_* and λ*_N_* correspond to the linear coordinates of the inner vertices *M* and *N* (unknown) along the detected Hough line *L_H_*. *C*_1_ corresponds to the center of projection of camera 1. Blue and green lines are outer and inner lines of sight (perspective lines), respectively. Π*_G_* represents the ground plane in a geo-referenced world coordinate system.

Since aerial images are employed, the proposed approach only deals with roof models due to the angle of view. Indeed, an aerial image allows the visualization of two facades at best, since the building generally has a rectangular footprint. Nevertheless, the building facades can actually be determined using the prior knowledge of the ground-height of the area under study (from urban database) and by the assumption that the dominant facade planes are vertical. In this paper, we restrict our study to simple polyhedral models (several roof varieties). Some are illustrated in Figure 5. The shown models present either horizontal and/or vertical symmetry assumptions and the inner and outer vertices respectively have the same height. These parametric building models with roofs having two, three, or four facets can also be described by a more generic building model (see Figure 6). In this model, any simple polyhedral model can be obtained by varying the 3D location of the inner vertices (*i.e.*, a deformable model) and by setting the height of all external vertices. Furthermore, the multi-facet model (Figure 6) and the one facet model (Figure 5(b)) can describe all typical situations: asymmetric shapes, sloping roofs or ground (*i.e.*, every vertex can have a different height). Hence, the proposed generic model describes more various building models than the model set shown in Figure 5. Since a complex building can be described as an aggregation of simple polyhedral building models, our approach can also deal with complex buildings once a partitioning of the building into simple building-parts is done. In this case, vertices are estimated for each simple model. The vertices having adjacent models are replaced by the barycenter of these points in order to reconstruct the final model.

The adopted multi-facet roof model comprises six vertices *A*, *B*, *C*, *D*, *M*, *N* (see Figure 6). In theory, the estimation of the roof model is equivalent to the estimation of the three-dimensional coordinates (*X, Y, Z*) of each vertex. As previously mentioned, the rectangular building footprint is manually selected in one image by an operator (interactive method). This footprint is considered as the footprint of reference for the succeeding processes. Moreover, the calibration of the aerial images is known (intrinsic and extrinsic parameters of the cameras). Consequently, the perspective 3D lines passing by the vertices of the image footprint are known. The 3D vertices that we seek to determine (*A*, *B*, *C*, *D*) are 3D points located along these perspective lines with unknown heights. In other words, by varying the height value, the corresponding 3D point slides along the perspective line. In this condition, the outer vertices we are searching for each have one degree of freedom. Hence, our polyhedral model can be simplified to ten parameters instead of eighteen: four parameters for the heights of the outer vertices and six parameters for the three-dimensional coordinates of the inner vertices. These ten parameters are encapsulated into one single vector **w**:

(1) $$w={\left(X_{M},\;Y_{M},\;Z_{M},\;X_{N},\;Y_{N},\;Z_{N},\;Z_{A},\;Z_{B},\;Z_{C},\;Z_{D}\right)}^{T}$$

Moreover, since the images are calibrated the 3D coordinates of the inner vertices *M* and *N* can be replaced by the triplets (*U_M_*, *V_M_*, *Z_M_*) and (*U_N_*, *V_N_, Z_N_*), respectively. (*U*, *V*) represent the image coordinates in the reference image.

Furthermore, it is easy to show that our polyhedral model can be fully described by the 3D coordinates of the inner vertices and of two outer vertices that are diagonally opposite (coplanarity constraint). Indeed, the building can be parameterized by eight parameters: four parameters for the image location of the inner vertices *M* and *N* but also four parameters for the height of the vertices *A*, *M*, *N*, and *C*. The remaining vertices are determined by intersecting the corresponding lines of sight with the estimated support planes. Indeed, we assume that *B* belongs to the estimated plane (*ANM*) and *D* belongs to the estimated plane (*CMN*) since the roof shape is supposed to be composed of planar facets. For these reasons, Equation (1) can be simplified to:

(2) $$w={\left(U_{M},\;V_{M},\;U_{N},\;V_{N},\;Z_{A},\;Z_{M},\;Z_{N},\;Z_{C}\right)}^{T}$$

where (*U_M_*,*V_M_* ) and (*U_N_*,*V_N_*) are the image coordinates of the vertices *M* and *N*, respectively.

In other words, our method has the obvious advantage that the coplanarity constraints are implicitly enforced in the model parametrization. By contrast, the feature-based approach requires fitting the planes to DSM or 3D points.

Recall that the 3*D* coordinates are expressed in a local coordinate system whose *Z*-axis coincides with the ground normal (the aerial images are geo-referenced). In practice, although the location of inner vertices is not known, the 2*D* line (the projection of a ridge segment) going through them can be easily extracted from the image by using a conventional edge detector (e.g., Canny edge detector) followed by a Hough transform. Once the equation of this line is known, the parametrization of the building model (Equation 2) can be further simplified to:

(3) $$w={\left(\lambda_{M},\;\lambda_{N},\;Z_{A},\;Z_{M},\;Z_{N},\;Z_{C}\right)}^{T}$$

where λ*_M_* and λ*_N_* parameterize the location of the inner vertices along the 2D segment obtained by intersecting the 2D line with the building footprint.

Thus, finding the model boils down to finding this vector **w**. Henceforth, we have defined the parametrization of the adopted generic building model. The succeeding section aims at describing a global methodology in order to determine the kind of model (one-facet or multi-facet) that corresponds to the reality as well as to compute the corresponding numeric parameters. To this effect, some computer vision mechanisms and strategies are described for 3D building shape recovery.

## 4. Proposed Approach

**Figure 7.**
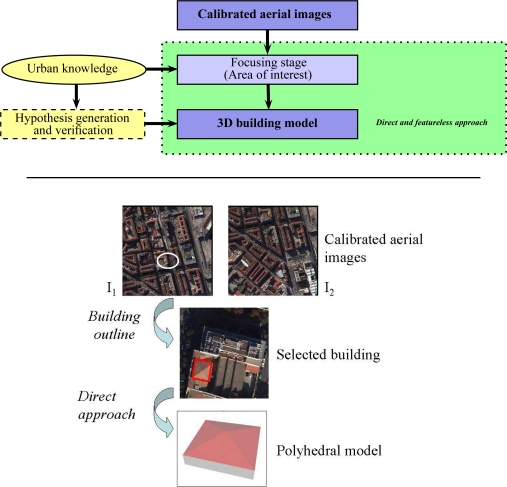
Flowchart diagram of the proposed approach (top) and illustrations of the main steps (bottom).

In this study, we present a novel modeling approach which is direct and image-based. The challenge consists in the reconstruction of 3D polyhedral building shapes using directly photometric information of aerial images. In computer vision, direct approaches have been essentially proposed for the image registration in order to generate mosaic images. Featureless image registration techniques strive to compute the global motion of the brightness pattern (e.g., affine or homographic transforms) without using matched features (e.g., [20]). We were inspired by this kind of approach and we propose a direct method for 3D building reconstruction. The flowchart diagram of the proposed approach is depicted in Figure 7.

### 4.1. Multiscopic Context and 3D to 2D Projection

As previously mentioned, our approach employs calibrated aerial images. The building under study is observed by *n* different points of view (*n* ≥ 2), in other words, in a multiscopic context. The visible area common to all the associated images is called overlapping area or overlapping volume. This area potentially characterizes the reconstructible area into 3D. We mention that geometric principles for 3D scene reconstruction from multiple views are described in detail in [35]. Besides, if the camera’s calibration is known and if the images are properly georeferenced, as assumed in our case, then an hypothetic physical 3D point *M*(*X, Y, Z*) (expressed in the world referential) that belongs to the overlapping volume can be projected in each image acquired by the camera *𝒞_i_* onto a corresponding image point *p_i_*(*u_i_*, *v_i_*) where 1 ≤ *i* ≤ *n*. These image points called homologous points can be calculated using a 3 × 4 projective camera matrix **P**:

(4) P=K·[R|T]

where **K** corresponds to the matrix of intrinsic parameters related to the camera *𝒞_i_*, **R** and **T** correspond to the extrinsic parameters which denote the coordinate system transformations from 3D world coordinates to 3D camera coordinates.

### 4.2. Measuring Model-to-Data Consistency

**Figure 8.**
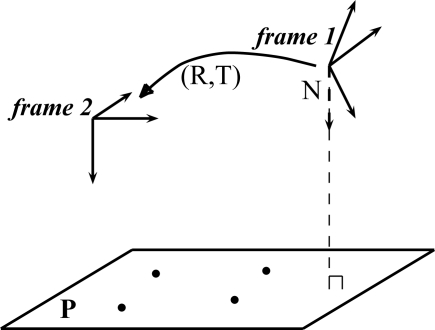
Homography induced by a plane.

In computer vision, the homography principle is employed in image registration, auto-calibration of cameras, motion estimation and also for stereoscopy and 3D scene reconstruction. Mathematically, the homography is a projective collineation that describes an image-to-image transformation that can be used either in the case of a pure 3D camera rotation, or a planar scene (see Figure 8). The homography matrix can be estimated by different techniques. An overview of these techniques are described in [19]. We are particularly interested by the homography principle since it can be used to transfer a roof facet of an image to another image if the 3D support plane of the facet is known.

The homography matrix is the transfer matrix that allows the transfer of the point *p*_1_(*x*_1_,*y*_1_) of the reference image (image 1) to its homologous point *p*_2_(*x*_2_,*y*_2_). The equation that links each pair of homologous points can be defined as:

(5) $$\left[\begin{array}{c} x_{2}\\ y_{2}\\ 1 \end{array}\right]≅\left[\begin{array}{ccc} H_{11} & H_{12} & H_{13}\\ H_{21} & H_{22} & H_{23}\\ H_{31} & H_{32} & H_{33} \end{array}\right]\left[\begin{array}{c} x_{1}\\ y_{1}\\ 1 \end{array}\right]\Leftrightarrow X_{2}≅{\mathrm{HX}}_{1}$$

where ≅ denotes the equality to a given scale factor. Equation (5) provides:

(6) $$\left\{ \begin{array}{l} x_{2}=\frac{H_{11}x_{1}+H_{12}y_{1}+H_{13}}{H_{31}x_{1}+H_{32}y_{1}+H_{33}}\\ y_{2}=\frac{H_{21}x_{1}+H_{22}y_{1}+H_{23}}{H_{31}x_{1}+H_{32}y_{1}+H_{33}} \end{array}\right.$$

The matrix **H** has eight degrees of freedom. For this reason, it is possible to judiciously select four points in another image in order to solve the system. We can note that this technique is generally employed with key points detectors. The coefficients of the matrix **H** depend on intrinsic and extrinsic parameters of the cameras as well as on the parameters of the plane:

(7) $$H≅K_{2}\;\cdot \;\left[R+\frac{T}{d}N^{t}\right]\;\cdot \;K_{1}^{-1}$$

where the matrix **K**_1_ and **K**_2_ respectively are the intrinsic matrix of the two cameras, **R** represents the rotation, **T** represents the translation vector (**R** and **T** represent the motion between the two cameras), **N** and *d* represent the parameters of the plane in the camera 1.

In our case, the intrinsic and extrinsic parameters of the cameras are known (calibrated cameras). The parameters of the plane need to be determined for each facet that compose the model. If the planes’ parameters are known, the homography matrix will directly transfer, facet by facet, sets of master pixels to their homologous pixels.

#### Measuring Facets-to-Data Consistency

In this subsection, a measure has been defined in order to value the accuracy of hypothetical facets according to the data. As we recall, in the multi-facet case, the facets are rigidly joined as shown in Figure 6. Firstly, we stress the importance of having a rigorous matching between the homologous points. The homologous points are 2D pixels representing the same 3D physical point in the scene. For this reason, the pixel intensities of homologous points have very close numeric values.

More precisely, our basic idea relies on the following fact: if the shape and the geometric parameters of the building (encoded by the vector **w**) correspond to the real building shape and geometry, then the pixel-to-pixel mapping (induced by homographies) between the master image *I_m_* (the one containing the selected 2D footprint) and any other aerial image (in which the building is visible) will be correct for the entire building footprint. In other words, the dissimilarity associated with the two sets of pixels should correspond to a minimum.

Recall that **w** is defining all support planes of all the building’s facets and thus the corresponding pixel *p*′ of any pixel *p* is estimated by a simple image transfer through homographies (3 × 3 matrices) based on these planes. Therefore, the associated global dissimilarity measure reaches a minimum. For an arbitrary model instance **w**, the global dissimilarity is given by the following score:

(8) $$e=\sum_{j=1}^{n-1}\sum_{p\in S}\rho \left(\left|I_{m}(p)-I_{j}\left(p^{\prime }\right)\right|\right)$$

where *n* is the number of aerial images in which the whole building roof is visible (in practice, *n* is between 2 and 5), *S* is the footprint of the building in the master image *I_m_*, *p*′ is the pixel in the image *I_j_* ≠ *I_m_* that corresponds to the pixel *p* ∈ *I_m_*, and *ρ*(*x*) is a robust error function.

The choice of the error function *ρ*(*x*) will determine the nature of the global error (8) which can be the Sum of Squared Differences (SSD) $\left(\rho (x)=\frac{1}{2}\;x^{2}\right)$, the Sum of Absolute Differences (SAD) (*ρ*(*x*) = *x*), or the saturated Sum of Absolute Differences. In general, the function *ρ*(*x*) could be any M-estimator [36]. In our experiments, we used the SAD score since it is relatively robust and its computation is fast.

We seek the polyhedral model ${\text{w}}^{★}={\left(\lambda_{M}^{★},\;\lambda_{N}^{★},\;Z_{A}^{★},\;Z_{M}^{★},\;Z_{N}^{★},\;Z_{C}^{★}\right)}^{T}$ that minimizes the above dissimilarity measure over the building footprint:

(9) $$w^{★}=\arg\;\underset{w}{\text{min}}\;e$$

We can also measure the fitness of the 3D model by measuring the gradient norms along the projected 3D segments of the generated 3D models. In general, at facet discontinuities the image gradient is high. Thus, for a good fit, the projection of the 3D segments will coincide with pixels having a high gradient norm in all images. Therefore, we want to maximize the sum of gradient norms along these segments over all images. Recall that we have at most nine segments for our simple 3D polyhedral model. Thus, the gradient score is given by:

(10) $$g=\frac{1}{n}\sum_{j}^{n}g_{j}$$

where *g_j_* is the gradient score for image *I_j_*. It is given by the average of the gradient norm over all pixels coinciding with the projected 3D model segments.

Since we want the dissimilarity measure (8) and the gradient score (10) to help us determine the best 3D polyhedral model, we must combine them in some way. One obvious way is to minimize the ratio:

(11) $$w^{★}=\arg\;\underset{w}{\text{min}}\;\frac{e}{g}$$

It is worth noting that during the optimization of (11) there is no feature extraction nor matching among the images. Furthermore, the use of the image gradient norms in (11) is not equivalent to a feature-based method.

The image-to-image transfer can be carried out pixel-to-pixel by combining 3D point construction (line of sight intersected with plane) and 3D-to-2D projection; or more directly facet-to-facet by using homographic transfer (Equation 5).

In order to minimize (11) over **w**, we will use an evolutionary optimizer that will be described in Subsection 4.3.

### 4.3. Computing the Polyhedral Building Model

In this subsection, we briefly describe the mechanisms and the goals of optimization processes. Moreover, we select an optimizer adapted to the considered modeling problem.

#### 4.3.1. Computing the Prismatic Building Model

In our case, our approach begins by approximating any building model by one horizontal facet, *i.e.*, adopting a prismatic model. An urban database is employed in order to know the minimum and maximum ground altitude *Z_ground_min_*, *Z_ground_max_* of the area under study as well as the minimum and maximum heights amongst all the included buildings *H_min_*, *H_max_* (e.g. *H_min_* = 5*m* and *H_max_* = 50*m*). The ground altitudes are provided according to the sea altitude. Consequently, we know with certainty that the building altitude *z* is in the interval *𝒤* = [*Z_ground_min_* + *H_min_*; *Z_ground_max_* + *H_max_*]. If we sweep this interval for *z* values with a step Δ*z*, then we obtain a set of candidates from which the best prismatic model is selected—the one that minimizes the objective function (11).

#### 4.3.2. Computing the 3D Model Using the Differential Evolution Algorithm

The Differential Evolution algorithm belongs to the family of Genetic Algorithms and to the evolutionary strategies. The genetic algorithm modifies the structure of individuals using the mutation and the crossover. The evolutionary strategies achieve the auto-adaptation by geometric manipulation of individuals. These ideas have been formulated by a simple and powerful operation of vectors mutation proposed in 1995 by Price and Storn ([37]). Since then the Differential Evolution has become an essential method for a large quantity of real problems and benchmarks.

The DE algorithm is employed in order to compute the 3D model and integrates the minimization process guided by the dissimilarity measure previously defined. This algorithm achieves generations of solutions—populations. The population of the first generation is randomly chosen around a rough solution. The rough solution will thus define a given distribution for the model parameters. The rough solution is simply given by a zero-order approximation model (the prismatic model) which is also obtained by minimizing the dissimilarity score over one unknown (the average height of the roof).

**Figure 9.**
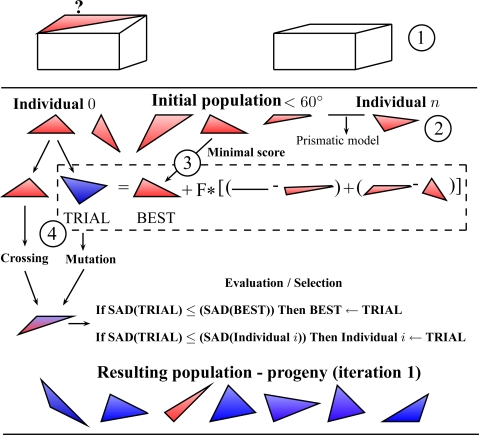
Illustration of one iteration of the DE algorithm.

In our case, the use of the DE algorithm is described in Figure 9 which illustrates the main steps performed in one single iteration for one facet. (1) The prismatic model is estimated, (2) a population is generated from the prismatic model, (3) the best model is determined, (4) a new solution is generated by using crossover, mutation and evaluation steps.

We use the Differential Evolution optimizer since it has four interesting properties: (i) it does not need an accurate initialization, (ii) it can integrate geometric constraints according to the context (adaptability properties), for example, constraints can be imposed in order to ensure that the polyhedral roof model are an assembly of facets with slopes inferior to 60° (standard information coming from urban databases concerning the area under study), (iii) it does not need the computation of partial derivatives of the cost function, and (iv) theoretically it can provide the global optimum. Hence, this algorithm is easy to implement and to integrate into the applications. In our case, the experiments show that only a few iterations lead to convincing results.

#### 4.3.3. Selecting One or Multi-Facet Building Modeling

**Figure 10.**
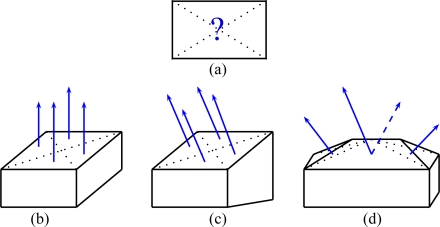
(a) Building footprint initially selected. No prior knowledge of the model shape is known. (b) Estimated prismatic model (algorithm initialization). (c) Estimated one-facet model (sloped roof). (d) Estimated multi-facet model (hip roof).

This subsection deals with the one-facet or multi-facet selection. Several automatic strategies can be employed:

- The first strategy consists in reconstructing the two models independently (one-facet model (sloped roof) and multi-facet model). Each calculated model provides a SAD score. The 3D model finally obtained will be the solution providing the minimum score among the models shown in Figures 10(b), 10(c), 10(d) in the sense that this score characterizes a better correspondence between the 3D model and the image data set.
- The second strategy (adopted) exploits the putative estimation of 4 facet normals. The building footprint (rectangular) is divided into 4 triangular facets as a pyramidal model (see Figure 10(a)). The estimation of the pyramidal model is driven by the evolutionary algorithm as described above. A geometrical constraint is imposed; namely, the central 3D point is located along the line of sight that passes across the central pixel of the footprint in the master image. In this first step, a pyramidal model is computed. In a second step, a score 𝒮_∠_ is set the highest deviation between the 4 facet normals and the vertical direction. If this computed score is less than a predefined tolerance threshold for normals verticalness denoted 𝒯_⊥_ (low angular deviation empirically fixed) then the retained model is the prismatic model initially calculated (e.g., Figure 10(b)). If this score exceeds this tolerance threshold (normals are non vertical), a score 𝒮′_∠_ is set to the highest deviation between the facet normals. If this calculated score is less than a predefined tolerance threshold for normals parallelism denoted 𝒯_‖_ then the one-facet estimation (e.g., Figure 10(c)) is carried out (estimation of a triangular facet using the rectangular footprint and estimation of the fourth vertex by intersecting the associated line of sight with the estimated plane). Otherwise, it means that the normal vectors are neither vertical, nor parallel (e.g., Figure 10(d)). In this condition, the multi-facet modeling is carried out.

It is worth noting that erroneous feature-based solutions as illustrated in Figures 4(a) and 4(b) (DEM-based) or coming from existing, less accurate, modeling pipeline could be used as initial solutions for the prismatic model estimation. Alternatively, they can also be used more directly as initial solutions for the Differential Evolution algorithm (multi-facet). In summary, the proposed approach proceeds in two parts. First, the algorithm decides if the building contains one or more facets. This decision is carried out by analyzing the 3D normals associated with four virtual triangles forming a partition of the whole building footprint. Second, once the model is selected, its associated parameters are then estimated by minimizing the defined dissimilarity score.

## 5. Experimental Results and Performance Study

In this section, we present the dataset employed as input of the proposed approach as well as the evaluations and the results obtained by our reconstruction method. We carry out several evaluations in order to analyze the convergence, the robustness and the accuracy of our image-based approach. These evaluations demonstrate the high potential of our modeling approach.

### 5.1. Input Dataset

**Figure 11.**
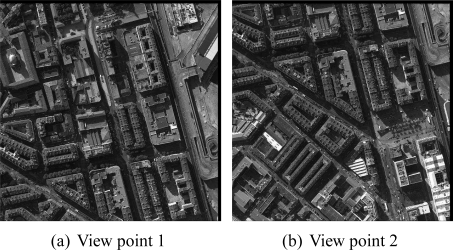
A pair sample of aerial images extracted of the multiscopic dataset. Each image covers a common area of the city of Marseille acquired from different points of view (partial overlapping). The size of the images is 𝒩*_c_* × 𝒩*_r_* = 4, 158 × 4, 160 where 𝒩*_c_* and 𝒩*_r_* correspond to the number of columns and rows, respectively.

The considered input dataset contains multiscopic gray-scale aerial images (see sample Figure 11). Each acquired image is described by a set of data specifying the georeferencing, the intrinsic parameters of the camera (e.g., focal, distortion coefficient, principal point) as well as the extrinsic parameters of camera location and orientation (rotation matrix, coordinates of point of view). These images have been acquired from an airplane, equipped with three cameras, coming from the aerial office of the city of Toulouse. A central camera was oriented vertically to the Nadir point. Two other cameras were mounted, one to the front of the plane and the other to the back of the plane, with a front and back oblique view. The resolution of the digital images is sub-metric and is around 10 centimeters. There are two key parameters related to acquisition, namely; 𝒝 that corresponds to the distance between two cameras (two positions) and 𝒣 that corresponds to the altitude of the flight. Generally, these two parameters are provided by the ratio $\frac{𝒝}{𝒣}$. A high ratio allows for a more accurate reconstruction. A low ratio allows a more reliable matching but reduces the accuracy. For the considered dataset, $\frac{𝒝}{𝒣}=\frac{230}{1280}\approx 0.18$. The acquired images are well-spaced and demonstrate a sufficient level of overlap. Hence, the input dataset is appropriate to carry out the 3D reconstruction.

**Comments:**
- The image resolution is sufficient in order to reach the intermediary level of modeling (Figure 1(b)).
- In order to reduce self-occlusion of roofs and perspective effects, we use images captured by the vertical camera only. Moreover, for a given set of Nadir images in which the building under study is visible, the master image is the one having its center the closest to the 2D building footprint. These choices for the camera and for the master image maintain a certain matching robustness.
- As previously mentioned, no Digital Elevation Model has been used in our approach.

### 5.2. Reconstructed 3D Models and Convergence Study

In this subsection, we measure the quality of the reconstruction obtained using the DE algorithm. We carry out a 3D modeling of one generic 3D facet running the DE algorithm with 30 iterations. The considered facet contains 5, 424 pixels. The number of individuals that compose the population is fixed to 30.

**Figure 12.**
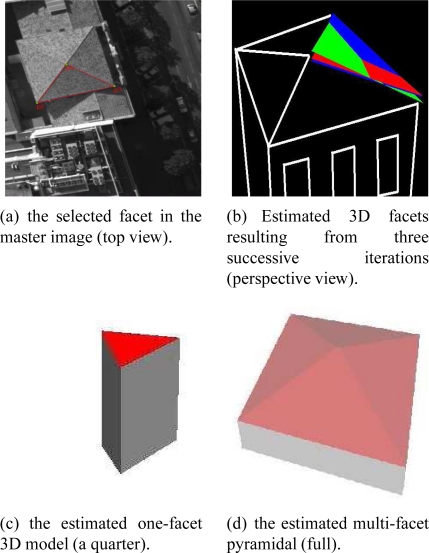
(a) illustrates the facet in the master image. (b) illustrates the successively estimated 3D facets during the evolution of DE algorithm. (c) and (d) illustrate the final estimated 3D model.

Figure 12(b) shows the estimated 3D facet corresponding to three iterations of the DE algorithm. The facets green, blue and red respectively correspond to the solution associated with iterations 1, 11 and 26, respectively. We can observe the facet evolved in the three-dimensional space. Each emerging facet represents a more accurate solution than the solution estimated in the preceding iteration. The intermediary solutions tend to correlate with the 3D ground truth. The image facet initially selected corresponds to a roof portion of a pyramidal building (Figure 12(a)). In Figure 12(b), the coarse structure drawn in white is only shown to accentuate the perspective effect for a better comprehension. The final 3D models (3D quarter and pyramidal model) resulting from the estimation are respectively shown in Figures 12(c) and 12(d).

**Figure 13.**
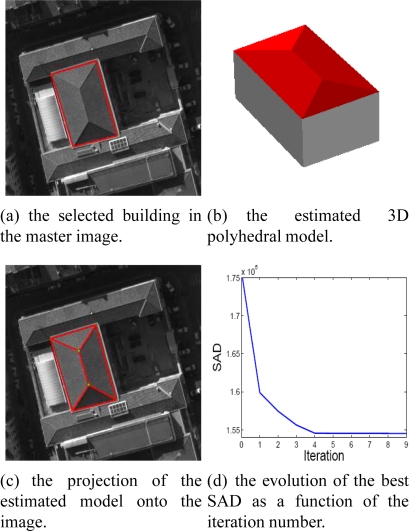
Estimated 3D polyhedral building model and related convergence.

Figure 13 shows the reconstructed 3D model (Figure 13(b)) associated with the building footprint selected in the master image (Figure 13(a)). The estimated 3D model projected into 2D images (e.g., 13(c)) enables the qualitative verification of the geometric coherence of the reconstructed 3D model. Figure 13(d) illustrates the global SAD score normalized by the number of pixels corresponding to the best individual obtained by each iteration. The graph evolution of the SAD shows the progressive evolution of the reconstructed 3D facets towards the ground truth configuration.

**Figure 14.**
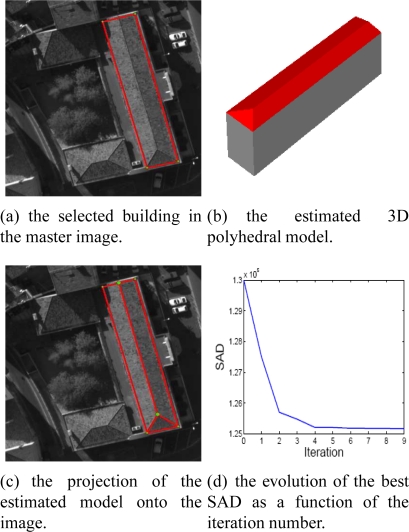
Estimated 3D polyhedral building model and related convergence.

**Figure 15.**
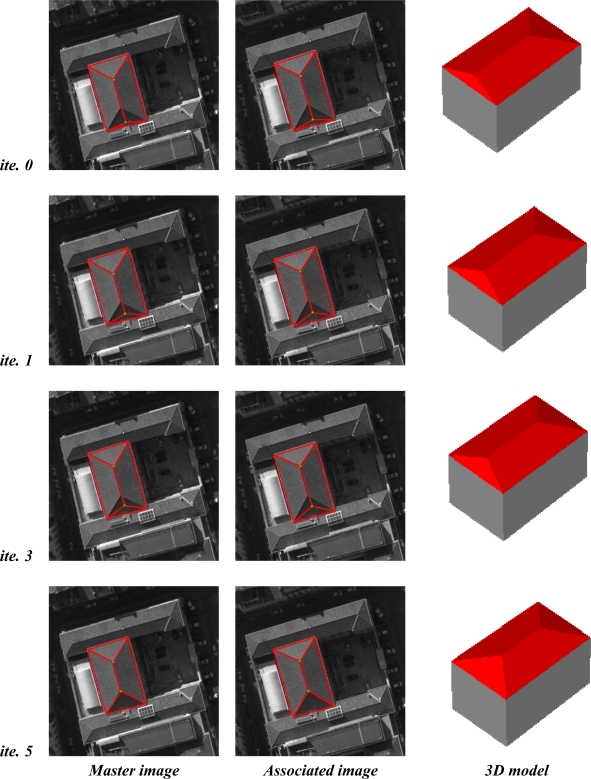
The best solution at several iterations of the Differential Evolution algorithm. The evolution of the 3D model and the footprint in the associated image is shown. The proposed algorithm converges to an optimal final solution in a few iterations.

Additional evaluations and results of reconstructed 3D building models and convergence are illustrated in Figures 14 and 15. In particular, Figure 15 shows in detail the evolution of a 3D polyhedral building model using the DE algorithm. The model convergence is analyzed in the aerial images (column 1 and 2) and in the three dimensional space (column 3) at different iterations of the algorithm (iterations 1, 3 and 5). We observe that the registration of the full building footprint (boundaries and inner line segments) between the master image (fixed footprint) and the other images (moving footprints) from the multiscopic dataset allows the correct 3D structure of the associated polyhedral model to be inferred. The registration process is guided by the DE algorithm. The estimated 3D models show a coherent converging shape from one to the next iteration. This continuity demonstrates the convergence reliability of the process.

### 5.3. Accuracy Evaluation

Figure 15 shows the results of reconstructing a building composed of four facets. The images shown in the middle column represent the projection of the estimated 3D model. At the end of the optimization process, we observe that the model points projected onto the other image coincide from one image to the other. This demonstrates that the estimated three-dimensional model is accurate. For a more quantitative evaluation, we selected one facet of this building and compared the 3D model with that obtained by a DEM-based modeling approach.

Table 2 provides a comparison associated with one 3D facet using on the one side, a DEM-based approach and on the other side, our proposed direct approach. The DEM-based approach from the known modeling pipeline previously mentioned has been used as a reference in our evaluation. Notice that the presented 3D facet solution (3D plane equation) has been estimated using a robust estimator by considering the complete set of 3D points included in the facet footprint.

The direct image-based and featureless approach provides a satisfying three-dimensional modeling since the results are very close to the ground truth data. As can be seen in Table 2, the results indicate an average deviation of around one decimeter.

Accuracy evaluation will also be studied in the following subsections that deal with the robustness evaluation of the proposed approach in complex cases.

### 5.4. Performance in the Presence of Image Noise

**Figure 16.**
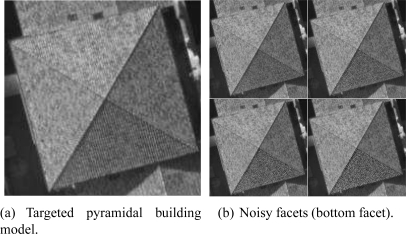
Adding noise to a facet for robustness evaluation. The intensities of the gray-scale pixels belong to the interval [0,255]. The magnitude 𝒨 of the uniform noise progressively increases according to the respective intervals 𝒨_1_ = [−4, 4], 𝒨_2_ = [−8, 8], 𝒨_3_ = [−16, 16] and 𝒨_4_ = [−32, 32]. These four levels of noise are shown in (b) (the corresponding random noise affected the bottom facet).

In order to get a quantitative evaluation of the 3D accuracy of the proposed approach, we adopted a simple and cheap scheme. For the sake of simplicity, we limited the study to a triangular facet that is viewed in two aerial images. In this scheme, we employ semi-synthetic aerial images (see Figure 16). Starting from a 3D facet model associated with a master image we synthesize the rawbrightness of this facet in the second image by simply warping its rawbrightness in the master image to the second image. The 3D selected solution will be considered as the ground truth. Each pixel belonging to the footprint in the second image is synthesized by its match from the master image using a bilinear interpolation and the corresponding ground-truth homography. In this case, the associated SAD value will be close to zero. We then add image noise to the transferred rawbrightness. The proposed reconstruction approach is then invoked in order to compute the 3D model of this facet. The deviation between the ground-truth 3D model and the estimated 3D model is calculated as a function of the noise magnitude.

**Figure 17.**
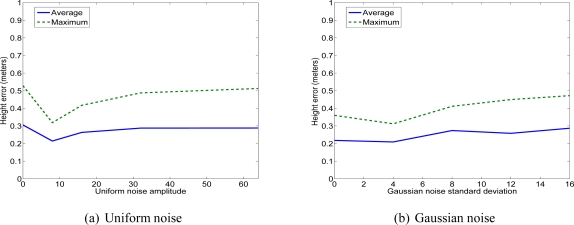
Error on the facet height.

We have used two kinds of image noise: uniform and Gaussian. The three first tests correspond to a uniform noise. The three succeeding tests correspond to a Gaussian noise (see Figure 17).

**Figure 18.**
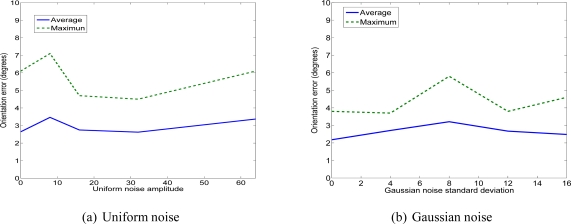
Error on the sloping angle.

**Figure 19.**
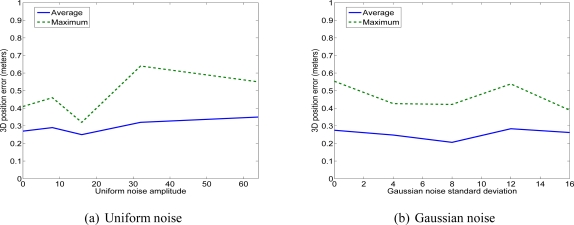
Error on the vertex 3D positions.

Figures 17, 18 and 19 show the errors of 3D reconstruction of one facet as a function of the level of noise, respectively uniform (to the left) and Gaussian (to the right). These errors are obtained by calculating an average over ten trials for each noise level, *i.e.*, ten reconstruction solutions. The 3D errors are expressed in meters. The *x*-axis shows the four levels of noise magnitude. The square deviation of the Gaussian noise is equal to 32 when the level of magnitude is equal to 4.

In this way, the added noise that progressively increases simulates images of buildings with different levels of quality and tests them for a 3D reconstruction. Thus, the first level of noise simulates slight defaults in the acquisition. In this way, we can test the robustness of our reconstruction method according to the quality of the acquired images.

We observe that the noise added to the image of footprints does not severely affect the accuracy of the 3D reconstruction. Depending on the type of noise, the average errors associated to the vertices can reach 33 cm, the average error associated with the sloping angle can reach 3.5° and the average error associated with the vertices altitude can reach 31 cm. Moreover, we can observe that the maximum errors have an inaccuracy multiplied by two to three with a maximum sloping angle of 7.2°, a maximum location deviation of 62 cm and a maximum altitude deviation of 53 cm. The values concerning the location and the sloping errors seem to oscillate while the altitude error seems to increase more with the noise. Nevertheless, despite the presence of high noise magnitude, the location deviations remain inferior to one meter and the sloping angle deviation is inferior to 10°. These values prove that the quality of the acquired images and their resolution are sufficient to allow accurate 3D building reconstruction using the proposed method.

### 5.5. Performance According to the Image Resolution

In this subsection, we propose to study the performance of the approach when the image resolution is reduced. A simple experiment was conducted. A triangular facet was selected. This facet contains 5432 pixels. We generated a sub-sampled facet by dropping every other column in the original image. Thus, we simulated a facet image with a reduced resolution.

Table 3 illustrates the 3D reconstruction of the tested triangular facet with the original resolution (first column) and with the reduced resolution (second column). The deviation between these two solutions is very small. The estimated 3D coordinates of the facet vertices globally varied for a few centimeters. In extreme cases, this deviation was around 20cm. In spite of a decrease in the image resolution, the accuracy of the estimated 3D models does not considerably downgrade.

### 5.6. Performance in the Presence of Superstructures

As previously mentioned, we aim to reconstruct planar roofs from aerial images. An important question comes to mind: what is the effect of superstructures on our reconstruction method? Indeed, a large majority of buildings incorporate superstructures. Consequently, the superstructures may generate unwanted noise since their 3D structures are not included in the dominant plane associated with the facet.

In this section, we present a method which increases the robustness of the facet reconstruction having several superstructures. The aim is to prevent the superstructures from distorting the estimation of the planar roofs. The idea consists in (i) detecting the pixels of the superstructures, and (ii) in using the footprint removed from these pixels. Assuming that the 3D plane calculated by the Differential Evolution algorithm is relatively accurate, we can thus classify the associated pixels into two categories: the pixels belonging to the dominant plane and the outlier pixels (pixels that do not belong to the plane). The proposed method proceeds in two passes:

- In the first pass, the DE algorithm is used with the totality of the reference footprint.
- In the second pass, the DE algorithm is used with only those pixels considered as belonging to the dominant plane.

Once the plane has been estimated in the first pass, several techniques can be used in order to carry out a coarse classification of the pixels. We observe that the pixels that do not belong to the theoretical plane of the facet will have a significant residual (absolute difference between the gray levels in different images) since the transfer pixel-to-pixel will not be correct. The idea is then to detect the pixels having a significant residual. We present then two techniques based on the threshold of individual residuals:

- The first technique selects the outlier pixels by determining a threshold for the residuals. The empirical threshold 𝒯_***emp***_ is defined as follows:
  (12) $${𝒯}_{\mathrm{emp}}=\mu +k\times \sigma$$
  where *k* is the coefficient of weight and has been manually determined. *μ* is the average of the individual residuals and *σ* is the associated square deviation.

For *k* = 0.2, we observe that the major part of the superstructures included in the facet (chimney and trap of roof) are detected.

- The second technique uses another formula for the threshold. This threshold noted 𝒯_***gen***_ is defined by:
  (13) $${𝒯}_{\mathrm{gen}}=2.5\times \mathrm{Me}$$
  where *Me* corresponds to the median value of the residuals associated with the whole facet footprint.

**Figure 20.**
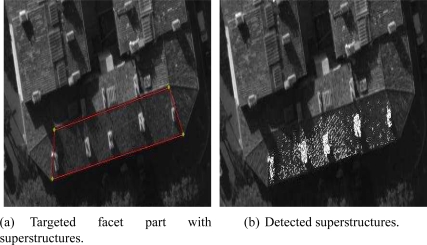
Automatic detection and filtering of the superstructures. The threshold 𝒯***_gen_*** is proportional to the median of all residuals. The removed pixels are shown in white.

**Figure 21.**
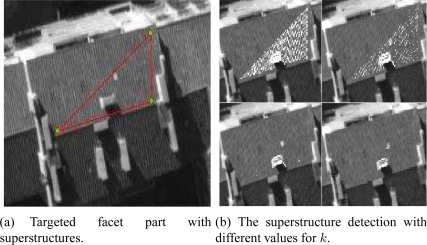
Filtering out the superstructures. (b) Tuning the *k* coefficient for the determination of the residual threshold 𝒯***_emp_*** (*k* = 0, *k* = 0.1, *k* = 0.2, *k* = 0.3, respectively, ). The removed pixels are shown in white.

We observe in Figure 20 that the major parts of the numerous superstructures belonging to the facet are detected. The detected white pixels shown in Figures 20(b) and 21(b) are ignored in the calculation of the final solution. All the pixels having higher residual values than 𝒯_***gen***_ are removed. The adopted threshold 𝒯_***gen***_ provides satisfying results for massive and generic filtering of the facet superstructures.

We have carried out a comparison of modeling methods by using the solution provided by the DEM as a reference solution. We have applied the reconstruction methods on one facet including superstructures namely several chimneys (see Figure 20(a)). The solution provided by the DEM has been obtained by estimating a 3D facet from all the associated elevation points via a robust estimator. Two measures of dissimilarity have been applied in our DE algorithm, in the first case, the SAD measure and in the second case, the SSD measure. Table 4 summarizes the 3D reconstruction of the facet using five strategies. We only report the height of the estimated vertices.

We observe the reconstructions to the altitude of the vertices since it is the parameter that varies the most (see Table 4). We have used the first technique in order to filter the superstructures. The coefficient *k* has been empirically tuned to 0.2. Although a part of the pixels belonging to the roof plane is filtered out, this part is low in comparison to the number of the considered pixels (several thousands) in the facet estimation.

We observe that the most accurate method seems to employ the SAD measure. Moreover, we find that the presence of superstructures can affect the 3D reconstructed model. A filtering stage is thus necessary in order to increase the accuracy of the solution. To this step, we envisage testing several methods integrating by different ways the superstructure filtering with the aim of model improvement. We stress on the fact that the DEM-based reference solution does not correspond to the ground truth.

### 5.7. Performance in the Presence of Significant Shadows

**Figure 22.**
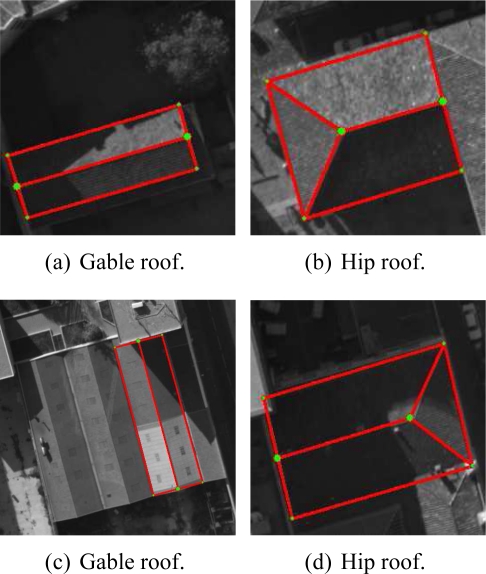
Correct building modeling in the presence of significant shadows. The master images are not shown.

As previously mentioned, the correct image registration of the building footprint leads to a correct 3D building model. Figure 22 illustrates the 3D modeling of some buildings using aerial images containing significant areas of shadow. In the figure, we visualize the 2D projection of the obtained 3D model in the non-reference image. As can be seen, for a variety of different buildings, the registration process is robust even in the presence of significant shadows.

## 6. Conclusions

### 6.1. Contribution

In this work, we provided an overview of some problems and solutions dealing with 3D building modeling. We proposed a new methodology for 3D building reconstruction based on a featureless process. To the best of our knowledge, this method has never been exploited in the 3D building modeling problem. Unlike existing methods, the pixel-to-pixel matching process is avoided. However, it is a by-product of the proposed method in the sense that once the 3D shape of the building is known, the image-to-image transfer is known from the associated homographies. The optimization associated with the proposed method has been carried out using the Differential Evolution algorithm. The method has been validated using real and simulated images. The proposed approach was compared with DEM based modeling approaches. It is beyond the scope of the current work to compare the proposed approach with all existing feature-based approaches. Indeed, it is well known that featureless approaches outperform feature-based approaches regarding the accuracy of the estimated geometric transforms used for image registration. The proposed method provides a satisfying polyhedral building reconstruction from gray-scale calibrated aerial images. The proposed top-down approach is also able to rectify erroneous reconstructed polyhedral building models in existing 3D city models whenever the corresponding aerial images are available. Furthermore, we argue that the proposed modeling method provides a novel tool that can be used in existing large-scale urban modeling pipelines as a main or complementary tool.

### 6.2. Future Work

Future work will be concentrated on the following directions:

- Testing other dissimilarity measures. New dissimilarity measures could be used and evaluated.
- Improving the reconstruction of roofs having superstructures. One possible solution is the integration of the outlier pixel filtering in the Differential Evolution algorithm. Indeed, each individual provides a normalized SAD score which considers only the pixels belonging to the roof plane. The pixels belonging to the superstructures will not be considered in the score calculation. The superstructure filtering will generate a different distribution in the progeny and provide a more accurate solution. The second scenario envisions running the proposed two passes several times.
- As we have previously mentioned, the registration process is carried out between a reference footprint (fixed boundaries) selected by an operator in the master image and the rawbrightness of the aerial images of the multiscopic data set in which the building is visible. Consequently, the initial boundary line segments of the reference footprint could be slightly shifted. In a future work, we intend to use a method that is able to deform a rough 2D footprint into a precise 2D footprint. This rectification process should precede the image based 3D reconstruction. We stress the fact that the use of cadastral maps can release the requirement of having an accurate 2D footprint.
- In our work, we assume that the building roof has conventional and simple shapes. Research could be done in the future in order to extend the direct approach to the case of generic buildings and roofs with atypical shapes.

## Figures and Tables

**Table 1.** Some feature-based approaches developed for 3D polyhedral building modeling from aerial images.

Paper	Process	Input data	Strategy
Jibrini *et al.*, [1]	Automatic	Urban map/Aerial Images	Bottom-up
Taillandier *et al.*, [2]	Automatic	Aerial Images	Bottom-up
Fischer *et al.*, [3]	Automatic	Aerial Images	Hybrid
Lafarge *et al.*, [8]	Automatic	Aerial Images	Top-down
Jaynes *et al.*, [14]	Automatic	DEM/Aerial Images	Hybrid
Zebedin *et al.*, [15]	Automatic	Aerial Images	Bottom-up
Tseng *et al.*, [21]	Interactive	Aerial Images	Top-down

**Table 2.** Comparison of 3D modeling results obtained in the first case from a DEM-based approach and in the second case from our direct image-based approach.

	DEM-based approach	Featureless proposed approach
*p*1	(117.59, 396.80, 26.95)	(117.66, 396.75, 27.21)
*p*2	(123.96, 387.79, 23.70)	(124.05, 387.74, 23.80)
*p*3	(108.36, 390.32, 24.51)	(108.33, 390.26, 23.85)
*Barycenter*	(116.70, 391.62, 24.97)	(116.66, 391.67, 25.06)

**Table 3.** Comparison of 3D modeling results in the cases original resolution and sub-sample images.

	Original resolution images	Sub-sampled images
*p*1	(117.65, 396.75, 27.14)	(117.65, 396.77, 26.87)
*p*2	(124.05, 387.75, 23.70)	(124.05, 387.75, 23.76)
*p*3	(108.35, 390.24, 24.44)	(108.34, 390.25, 24.27)

**Table 4.** Comparing the modeling results obtained with the SAD and SSD scores using facets including superstructures with and without the filtering process.

	DEM	SAD	SAD	SSD	SSD

Including		superstructures	filtering	superstructures	filtering
*z*1	41.96 m	42.92 m	42.22 m	43.61 m	42.75 m
*z*2	41.36 m	41.10 m	40.98 m	40.84 m	40.87 m
*z*3	39.78 m	39.62 m	40.22 m	38.88 m	40.10 m
Average deviation in *z*	0.0 m	0.46 m	0.36 m	1.02 m	0.53 m
